# Exploitation of Skin Microbiota in Wound Healing: Perspectives During Space Missions

**DOI:** 10.3389/fbioe.2022.873384

**Published:** 2022-04-29

**Authors:** Massimiliano Marvasi, Monica Monici, Desirée Pantalone, Duccio Cavalieri

**Affiliations:** ^1^ Department of Biology, University of Florence, Florence, Italy; ^2^ ASAcampus Joint Laboratory, ASA Res. Div., Deptartment of Experimental and Clinical Biomedical Sciences “Mario Serio”, University of Florence, Florence, Italy; ^3^ Emergency Surgery Unit-Trauma Team, Emergency Department, Department of Experimental and Clinical Medicine, Careggi University Hospital, University of Florence, Florence, Italy

**Keywords:** microbiome and dysbiosis, wound healing, space mission, skin, wound

## Abstract

Wound healing is slowed in Space. Microgravity and possible physical factors associated with Space affect alterations in fibroblast, matrix formation, dysregulation in apoptosis and inflammation. The microbial populations settled on skin, space modules, in space suits, are also playing a pivotal role, as wound healing is also affected by the microbial community. We propose a perspective that includes four domines for the application of human skin microbiota for wound healing in Space: The natural antimicrobial properties of the skin microbiota, the crosstalk of the skin microbiota with the immune system during wound healing, the contribution of the microbiota in precision medicine, and the role of gut-skin and gut-brain axes. A stronger understanding of the connections and metabolic network among bacteria, fungi, the host’s immune system and the host metabolism will support the basis for a better wound healing in Space.

## Introduction

Wounding can happen during Space exploration (Gontcharov et al., 2005; Tronnier et al., 2008; Crucian et al., 2016; Farkas and Farkas, 2021). Astronauts are exposed to skin erythema, peeling, dryness, burning, pruritus, sensitivity, thinning, therefore the physiology of the healing process of cutaneous injuries is hence affected (Farahani and DiPietro, 2008; Cubo-Mateo and Gelinsky, 2021). Recent studies have confirmed that wound healing is altered during Space missions (Cubo-Mateo and Gelinsky, 2021). During the normal wound healing process and under normal gravity (on earth), the body can repair wounds, trauma, burns by recreating the skin barrier (Reinke and Sorg, 2012; Singh et al., 2017; Maheswary et al., 2021). The physiology of wound healing under normal conditions is achieved *via* several defined steps: inflammation, proliferation, epithelialization, maturation, and remodelling phases (Reinke and Sorg, 2012; Singh et al., 2017; Maheswary et al., 2021). Chronic wounds extend the inflammatory phase, with immune cells continually degrading collagen and extracellular matrix (Blaber et al., 2010; Dam and Paller, 2018; Jemison and Olabisi, 2021). The delayed wound healing induced by microgravity differs in aetiology from that of chronic wounds on earth (Jemison and Olabisi, 2021). Microgravity affects matrix formation (Blaber et al., 2010), the alterations in fibroblast and dysregulation in apoptosis (Cialdai and Monici, 2013), inflammation (Blaber et al., 2010), delayed cellular proliferation of the basal skin layer and a thinning of the upper layer of the epidermis (this study is based on a single crew member) (Tronnier et al., 2008). Space radiation including solar particle events, galactic cosmic radiation and intra-vehicular secondary radiation may also compromise the physiology of the skin (Onorato et al., 2020). Space radiations cause damage to the DNA, through direct interaction or production of free radicals (Moreno-Villanueva et al., 2017; Afshinnekoo et al., 2020). Non-ionizing UV radiations also damage the skin as well, but this is not an issue in Space due to extensive protection provided to the astronauts (Tyrrell, 1995; Gasperini et al., 2017; Lipsky and German, 2019). UV exposure is under strict control inside the International Space Station (ISS) also in case of use of UV lamps.

Physical factors are not the only factors that affect the physiology of skin. The microbial populations settled on skin, space modules, in space suits, are also playing a pivotal role. The skin hosts an immense number of microorganisms adapted to utilize the nutrients available. The skin microbiota of healthy adults remains stable over time, despite environmental perturbations—at least on earth (Byrd et al., 2018). It is well known that skin diseases and disorders, are associated with an altered microbial state (Lunjani et al., 2019). Space missions occur in non-sterile, extreme confined environments, where air pressure, temperature, humidity, limited water supply are kept under strict control (Gentry and Cover, 2015). In this environment, astronauts are not able to take proper shower keeping their body clean by using wet tissues, using rinseless shampoo, and they do not change their clothes often (Farkas and Farkas, 2021). It is therefore important to understand the astronauts’ skin microbiomes and their fluctuation over time.

The skin microbiota has been studied by using both cultivable microbes and metagenomic profiles. The application of targeted and untargeted Next Generation Sequencing (NGS) has been used to further differentiate strains and functional variability (Tomida et al., 2013), e.g., the association of microorganisms with sweet and sebaceous glands, hair follicle, sebum and the stratum corneum (Byrd et al., 2018). Bacteria may variate according with the site of the body, in contrast fungal community composition was in general similar across core body sites regardless the physiology (Findley et al., 2013; Byrd et al., 2018).

The ISS is not a sterile environment and its microbial community is routinely monitored on various surfaces, as part of standard operations and maintenance requirement procedures (NASA, 2005). Influx of new microbes from travels at the ISS may quickly resemble astronaut skin microbiomes: it is transient (following crew exchanges), and some can settle permanently (Voorhies et al., 2019), showing that there is shift of the microbial communities, but also a small proportion of ubiquitous bacteria are long-term residents (in dust, Actinobacteria, Proteobacteria, and Firmicutes) (Checinska et al., 2015). A survey of cultivable bacterial and fungal populations from surface wipes over 14 months from the ISS surfaces, showed a range of 10^4^–10^9^ CFU/m^2^ changing over time but remained similar between locations. With reference to the phyla, the bacteria Actinobacteria, Firmicutes, and Proteobacteria were the most represented, while Ascomycota and Basidiomycota phyla represented the fungal domine. The dominant organisms are associated with the human microbiome and may include opportunistic pathogens. Methylobacteriaceae were also dominant across the ISS (as well as in some hospitals) (Checinska Sielaff et al., 2019), and Staphylococcaceae and Enterobacteriaceae were the most predominant organisms on the US module, very similar to fitness centres and, again, hospitals (Mukherjee et al., 2014; Lax et al., 2017). Interestingly, 46% of viable bacteria and 40% of viable fungi from the overall meta-taxonomical 16S were culturable, reflecting that a high percentage of possible opportunistic pathogens are present and alive, suggesting that ISS is like other built environments (Checinska Sielaff et al., 2019). Recent findings suggest that possible thinning of the upper layer of the epidermis and a significant loss of elasticity of the skin (Tronnier et al., 2008) could increase the exposure of the microbial communities that reside in the deeper layers of the skin, including the stratum corneum (Zeeuwen et al., 2012; Lipsky et al., 2020). In this conditions the skin of astronauts is continuously exposed to different microbial communities with relatively low biodiversity (Checinska Sielaff et al., 2019; Voorhies et al., 2019).

A crosstalk in Space: application of the knowledge on skin microbiota during wound healing.

The skin microbiome is protective against pathogens, nevertheless in certain conditions the microorganisms that are ordinarily beneficial to their host can become pathogenic (dysbiosis) (Byrd et al., 2018). Skin microorganisms are important in educating the innate and adaptive arms of the cutaneous immune system (Byrd et al., 2018), and the commensal skin microbiome during healing is essential for the regulation of the cutaneous immune system (Tomic-Canic et al., 2020). Skin microorganisms can provide protection from pathogens through modulation of antimicrobials (Tomic-Canic et al., 2020). It is urgent to better understand the crosstalk of the skin microbiota and the immune system in Space, as it is difficult to disentangle the components of microgravity, space radiations, stress, and the effect of the microbiota itself (Siddiqui et al., 2021). Exposure to microgravity during Space flights produces immunosuppression, and this can lead to dysbiosis or ill-states (Spatz et al., 2021).

### The Skin Microbiota has Natural Antimicrobial Properties

**FIGURE 1:**
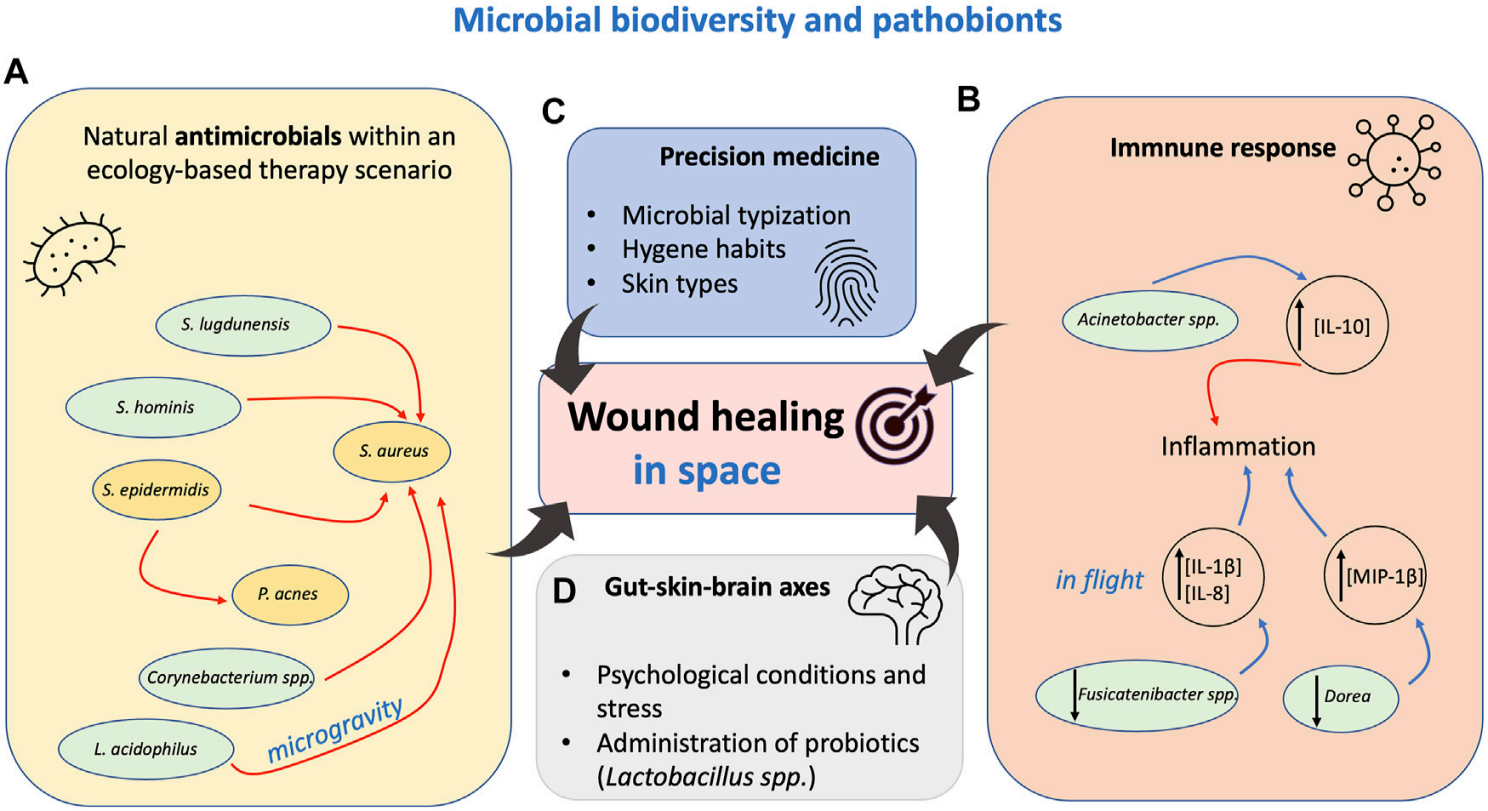
Perspectives for the application of human skin microbiota for wound healing in Space. Four possible domines for intervention developed from studies aimed to improve medical applications on Earth. These solutions can also find application in Space. **(A)** The ecological network among microorganisms is also regulated by natural antimicrobial. Few examples are shown as reported in the main narrative. *S*, *Staphylococcus* spp. *L*, *Lactobacillus. P, Propionibacterium.* Red arrows represent inhibition. Gold boxes are common pathobionts. Green boxes are other organisms. **(B)** Crosstalk of the skin microbiota with the immune system during wound healing. Microorganisms can support cytokine interleukin regulation. Up and down black arrows mean activation and reduction, respectively. Blue arrow represents IL expression. **(C,D)** The typization of the skin microbiota can lead to a better treatment of wound and prevention of complications. Gut-skin, gut-brain axes can be crucial for healing: in mice the administration of probiotics has shown better regulation of healing. Please refer to the text for all the details.

The role of microorganisms in chronic wounds provides important insight on how to handle would healing in Space (Figure 1). The role of the microbiome in chronic wound is not fully understood and the importance of topical antimicrobial agents in their treatment is continuously debated (Tomic-Canic et al., 2020). Pathogens or pathobionts are suspected to delay the healing process (Eming et al., 2014; Misic et al., 2014). Alterations in the skin microbiome might contribute to the high frequency of skin rashes/hypersensitivity episodes experienced by astronauts in Space (Voorhies et al., 2019). Microorganisms can act competitively to exclude one another or synergistically for mutual benefits depending by the interaction between the skin surface and other microorganisms that live on it (Cleary et al., 2018).

Microgravity can affect the biology of microorganisms. Under simulated microgravity, the liquid phase of a culture of *Lactobacillus acidophilus* exhibited higher antibacterial activity against *Staphylococcus aureus* in a time-dependent manner (Shao et al., 2017). Targeting *S. aureus* and *Staphylococcus epidermidis* in wounds is important. *S. epidermidis* can either help or hurt the skin barrier being strain-dependent in disease (Brown and Horswill, 2020). *Staphylococcus lugdunensis* inhibited *S. aureus* growth through the production of the antibiotic lugdunin, a thiazolidine-containing cyclic peptide (Zipperer et al., 2016). In another study, *S. epidermidis* strains were capable of inhibiting *Propionibacterium acnes* grown *in-vitro* (Christensen et al., 2016). *P. acnes* is involved in developing cutaneous inflammation. The peptidoglycan of *P. acnes* can activate monocytes to produce cytokines such as IL-1β, IL-8, and TNF-α, which cause granulomatous responses in inflammatory skin disorder (Kistowska et al., 2014; Lee et al., 2019).

In a different study, multiple coagulase-negative *Staphylococcus* spp, *S. epidermidis* and *Staphylococcus hominis* were shown to produce lantibiotics that were able to synergize with the human antimicrobial peptide cathelicidin and to inhibit the growth of *S. aureus* (Nakatsuji et al., 2017; Byrd et al., 2018). Strains producing lantibiotics were depleted in individuals with atopic dermatitis, who are frequently colonized with *S. aureus* (Byrd et al., 2018). Other studies have shown that *S. aureus* shifts toward commensalism in response to *Corynebacterium* species, affecting *S. aureus* behaviour and fitness, leading to a reduced virulence of *S. aureus* (Ramsey et al., 2016). *In vitro* mono-vs. co-culture of commensal *Corynebacterium striatum* increased transcription of genes related to human nasal colonization and decreased transcription of virulence genes of *S. aureus* (Ramsey et al., 2016)*.* Biotechnological application of *Corynebacterium*’s metabolic products could lead to develop anti-virulence therapies against *S. aureus* (Ramsey et al., 2016).

It must be mentioned that the direct influences of changes of the microbial community should be carefully considered, since changes in abundance may not be solely beneficial but leading to opposite results, For example, *Propionibacterium* species induced *S. aureus* aggregation and biofilm formation in a manner dependent on dose, growth phase and pH (Wollenberg et al., 2014).

The discovery, characterization and production of the antimicrobials or elicitors involved in quorum sensing or signalling could find important biotechnological applications to control pathobionts. The interactions among microorganisms in wounds are common but poorly characterized.

Bacterial community instability was associated with faster healing and more positive clinical outcomes (Loesche et al., 2017). One hundred subjects diagnosed with diabetic foot ulcers were enrolled into a prospective, longitudinal cohort study to analyse the temporal dynamics of the bacterial community of the ulcers. In this study the bacterial community stability reflected a delayed healing phenotype (Loesche et al., 2017). This observation may apparently be counterintuitive, as in many other pathologies bacterial community instability was associated with disease (Martinez et al., 2008). Nevertheless, as proposed by Loesche and collaborators “instability in the microbiome is a reflection of effective control of wound bacteria, which prevents any community structure from stabilizing” (Loesche et al., 2017). Stabilization could proceed on the trajectory of the progressive ulceration. Temporal stability of the microbiota of wound should be further studied in Space.

In another similar study, the mycobiome (the fungal microbial community) in chronic wounds is predictive of healing time, associated with poor outcomes when forming mixed fungal-bacterial biofilms (Kalan et al., 2016). *Cladosporium herbarum* and *Candida albicans* have been identified as the most abundant species in chronic wounds (Kalan et al., 2016). More information must be acquired from the microbiome of chronic wounds, which is believed to play an important role in impaired healing and the development of infection-related complications (Loesche et al., 2017). It is not known to what extent the mycobiome composition in Space provides little predictive value of wound outcomes, and culture-independent studies have been limited by cross-sectional design and small cohort size. An interesting approach adopted by Loesche and collaborators was to define the outcome of the diabetic foot ulcers by classifying the microbiota in different community types (Loesche et al., 2017). In this disease the transition patterns and frequencies of microbial populations were associated with healing time, and this can be extended to the prognosis. Similar experimental approaches could be done in microgravity scenarios.

### Crosstalk of the Skin Microbiota With the Immune System During wound Healing

The immune system is interconnected with the skin microbiota, especially by targeting pathogen-associated molecular patterns (PAMPs), through pattern recognition receptors (PRRs). For example, *Propionibacterium acnes* and the lipopolysaccharides induce the expression of antimicrobial peptides and proinflammatory cytokines/chemokines in human sebocytes contributing to the host defence and skin inflammation (Nagy et al., 2006; Naik et al., 2012). Microorganisms could be beneficial if able to regulate the immune response toward a normal healing process. For example, the lack of interleukin-10 (IL-10) (a key anti-inflammatory cytokine in immunologic tolerance) results in a strong inflammatory response. IL-10 is a key mediator of the pro-to anti-inflammatory transition that counters collagen deposition (Singampalli et al., 2020). In healthy individuals, IL-10 expression was positively correlated with the abundance of the gammaproteobacterial genus *Acinetobacter* on the skin (Hanski et al., 2012) (Figure 1). Abundance of *Acinetobacter* could be monitored in astronauts, and further biotechnological elicitors released by *Acinetobacter* could be used as food additive to support wound healing in microgravity, as well as on earth.

It is well known that environmental biodiversity, human microbiota, and allergy are correlated (Hanski et al., 2012). Pro-inflammatory cytokines MCP-1, IL-8, IL-1b and MIP-1β showed a significant increase in their concentration during flight (flight day, FD 180), while TNFa had a near significant rise. Conversely, a decrease in OTU of *Fusicatenibacter* spp. and *Dorea* spp. was measured when IL-8, IL-1 β and MIP-1β increased during flight (Figure 1). Cytokine concentrations reverted to pre-flight levels within 2 months of returning to earth (Voorhies et al., 2019). It would be interesting to further understand the association of the astronauts’ immune dysregulation with the skin microbiome. The main picture is still far to be resolved. The analysis of skin microbiota forehead and forearm (Voorhies et al., 2019) (Voorhies et al., 2019) (Voorhies et al., 2019) (Voorhies et al., 2019) during inflight showed a significant reduction of Proteobacteria, mainly, Gamma and Betaproteobacteria, with an associated increase in Firmicutes, including Staphylococcal, and Streptococcal species (Voorhies et al., 2019) (Figure 1). A better regulation of Proteobacteria on skin could also provide further details on how the dynamics of the microbiota can regulate pro-inflammatory cytokines and wound healing in Space. Voorhies and co-authors proposed that it is possible that the constant filtration of environmental air in the ISS contributes to the overall reduction of skin Proteobacteria (Voorhies et al., 2019). Recent studies conducted in children showed the importance of “green” areas around the homes. Children living nearby green areas showed reduced atopic sensitization, the same absence of “green” resources (possibly “green” areas) could reduce Proteobacteria on the skin to a pathological level (Ruokolainen et al., 2015). Absence of “green” resources (or better, reservoir of similar microbial communities) could control the incidence of skin clinical symptoms during long-duration orbital spaceflight (Crucian et al., 2016; Voorhies et al., 2019). It is reasonable to assume that skin clinical symptoms and changes in skin structure may facilitate the establishment of skin infections, inflammation, leading to reduced wound healing during spaceflight. Further research needs to be addressed.

### Precision Medicine, Personal Skin Features in Wound Healing

The microbial typing for each individual is ideal for precision medicine approaches, leading treatment of wounds (Hülpüsch et al., 2021) (Figure 1). This important field of research needs to be further explored in Space: it would be important to understand to what extent the skin microbiota changes in astronauts according with each personal skin moisture, pH, personal hygiene habits. It is not clear how these factors affect wound healing and it would need further investigations (Voorhies et al., 2019). In this context it would be interesting to stimulate *ad hoc* skin probiotic communities in an ecology-based therapy scenario, by limiting dysbiosis that leads to cutaneous disorders (Zhou et al., 2020).

### Gut-Skin, Gut-Brain Axes can be Crucial for Wound Healing in Space

The crosstalk between the immune system and the skin microbiota can lead to normal healing (Byrd et al., 2018). In this context the gut-brain/skin axis can play a role in wound healing (Figure 1). The gut microbiome influences many domains of the human body: e.g., the central nervous system (Ma et al., 2019), endocrine control (Régnier et al., 2021), immune fitness (Balikji et al., 2021). New biotechnological approaches should identify microbial elicitors that could be used to control inflammatory cytokines and be administered as probiotics or as additive in food. Probiotics actively can crosstalk between the immune system and the skin microbiota. From the biotechnological potential a few examples have been proposed: in mice, the administration of *Lactobacillus reuteri* enhanced wound-healing properties through the up-regulation of the neuropeptide hormone oxytocin (Poutahidis et al., 2013). In mice, diet control has been proved to alter the formation of chronic wounds, as a diet with kefir products or the administration of *Lactobacillus johnsonii* showed to support some benefits (Guéniche et al., 2006; Huseini et al., 2012). In another murine model, preparations of orally administered *Lactobacillus acidophilus*, showed enhanced wound contraction and fast epithelization when compared with the not treated. The treatment with *L. acidophilus* also increased breaking strength in sutured incision wound, increased granuloma dry weight and marked increase in collagen content indicating wound healing (Gudadappanavar et al., 2017). The use of *Lactobacillus* spp. in food biotechnology is well known, it has essentially negligible biological risk, and it is known for balancing gut microbial population (Bernardeau et al., 2006; Widyastuti et al., 2021).

It is reasonable to assume that the gut-skin and gut-brain axes biochemical signalling could also affect wound healing in Space, having a profound influence on astronaut health (Siddiqui et al., 2021). Further studies must be completed. For example, depression and chronic wounds were demonstrated to share common pathologic features, which included dysregulated inflammation and altered microbiome (Hadian et al., 2020). For this reason, it should not be excluded that isolation and stress associated with the fluctuation of the microbiome can originate delay in healing. Recently, in a questionary survey about the irritable bowel syndrome symptoms, the perceived immune fitness (subjective judgment), and impaired wound healing were studied in a cohort of 1942 Dutch students (Balikji et al., 2021). The assessment showed that impaired wound healing (self-reported) was significantly associated with irritable bowel syndrome, showing that when both conditions occurred, complaints were significantly more severe (Balikji et al., 2021).

## Conclusion

The last frontiers on the association of the skin microbiome and wound healing during short and long mission in Space show that further understanding is needed. The perspectives for further research range from studies on microbial dynamics, natural antimicrobial properties, understating how personal skin features affect healing, the crosstalk of the microbiome with the immune system, to the skin-gut-brain axes.

Further biotechnological applications will help to understand better the skin microbiome and the healing process: for example, the three-dimensional bioprinting of skin utilizing different cell types (fibroblasts and keratinocytes) as bio-inks for tissue engineering (Jemison and Olabisi, 2021). This technology can help in studying the healing process associated with specific probiotic microbial communities (mediated by elicitors), or the deposition of different cell types along with specific biomaterials (Cubo-Mateo and Gelinsky, 2021). This is particularly important for travels to Mars, where also surgery can be a possible scenario (Kirkpatrick et al., 1997; Farahani and DiPietro, 2008; Cubo-Mateo and Gelinsky, 2021).

In this context, human skin-associated microorganisms provide a pivotal role to the skin-microbe ecosystems that still need to be fully understood. A stronger understanding of the connections and metabolic network among bacteria, fungi, the host’s immune system and the host metabolism will support the basis for a better wound healing in Space. Improving our knowledge in this field of the biomedicine not only can be important in the perspective of ensuring adequate medical assistance in future space exploration missions beyond LEO, but it can have important repercussions on earth. In fact, chronic ulcers, which are continually at risk of infection, are a huge health problem, with high social costs and poor quality of patients’s life.

## Data Availability

The original contributions presented in the study are included in the article/Supplementary Material, further inquiries can be directed to the corresponding author.
